# Three-Dimensional-Derived Echocardiographic Left Ventricular Structure and Function and Indices from the 12-Lead Electrocardiogram across the Menstrual Cycle in Healthy Physically Active Females: An Exploratory Study

**DOI:** 10.3390/jcdd10080331

**Published:** 2023-08-03

**Authors:** Barbara N. Morrison, Allison J. Campbell, Anita T. Coté, Aleah Mohammad, Laura Sambrook, Georgia Robinson, Keith George, David Oxborough

**Affiliations:** 1 School of Human Kinetics, Trinity Western University, Langley, BC V2Y 1Y1, Canada; barbara.morrison@twu.ca (B.N.M.); allison_campbell_5@sfu.ca (A.J.C.); anita.cote@twu.ca (A.T.C.); 2 Research Institute for Sport and Exercise Sciences, Liverpool John Moores University, Liverpool L3 3AF, UK; mohammadaleah@gmail.com (A.M.); laura.sambrook2@nhs.net (L.S.); georgialucie@hotmail.com (G.R.); k.george@ljmu.ac.uk (K.G.)

**Keywords:** menstrual cycle, eumenorrhea, echocardiography, electrocardiogram, left ventricular function, left ventricular structure

## Abstract

**Background**: The impact of the menstrual phases on left ventricular (LV) structure and function using 3D echocardiography and resting electrocardiogram (ECG) in healthy, eumenorrheic, and physically active females has not been investigated. **Methods:** sixteen females (20 y ± 2) underwent 3D echocardiography and an ECG at three time points in the menstrual cycle phases (follicular, ovulation, luteal). LV end-diastolic volume (LVEDVi), LV ejection fraction (LVEF), LV mass allometrically indexed to height^2.7^ (LVMi), torsion, and global longitudinal, circumferential, and radial strain (GLS, GCS, and GRS) were evaluated. ECG data of the P and QRS waves were presented as well as axis deviation, chamber enlargement, and any rhythm abnormalities. **Results:** LVMi was significantly higher in the luteal phase (36.4 g/m^2.7^ ± 3.3) compared to the follicular (35.0 g/m^2.7^ ± 3.7) and ovulation (34.7 g/m^2.7^ ± 4.3) phases (*p* = 0.026). There were no differences in other indices of LV structure and function or ECG variables across all phases of the menstrual cycle or evidence of arrhythmia. **Conclusions:** In physically active females, there is a small but significantly higher LVMi associated with the luteal phase of the menstrual cycle with no concomitant change in LV function or ECG parameters. These findings are important to consider when conducting clinical or research serial assessments.

## 1. Introduction

During the menstrual cycle, the concentrations of estrogen and progesterone fluctuate and have pleiotropic effects on the cardiovascular system [1]. For example, estrogen levels can rise to 2000 pmol/L prior to ovulation, before falling to 100 pmol/L during menstruation [2]. These cyclical changes in hormonal concentration throughout the menstrual cycle may affect cardiac structure and function [1]; however, the direct impact on left ventricular (LV) structure and function remains unclear [3,4,5,6]. Previous studies have predominately used conventional echocardiography to assess global structure and function; however, these measurements are limited by an inability to detect subtle changes in function [7,8]. For example, previous research has demonstrated that LV mass (LVM), wall thickness, LV end-diastolic volume (LVEDV), and LV end-systolic volume (LVESV) as determined by two-dimensional (2D) echocardiography are unchanged throughout all stages of the menstrual cycle [3,4,6]. This is in contrast to other studies that have reported a phasic increase in LV systolic and diastolic function using conventional indices [4,9]. The lack of consistency in the data may be related to a heterogeneous presentation of the population and/or inherent limitations of conventional echocardiographic assessment. The extent and nature of these fluctuations are important to understand when conducting serial assessments such as during cardiac pre-participation screening, repeated measures research studies, or routine clinical visits. 

Although assessment of LV structure and function is conventionally made using 2D echocardiography, LV volume and LVM from 3-dimensional (3D) echocardiography have been shown to be more accurate and reproducible and correlate with the current gold standard of cardiac magnetic resonance imaging (CMR) [10,11]. Additionally, 3D echocardiographic post-processing software uses volumetric analysis, avoiding the need to use geometric assumptions [10]. Speckle tracking echocardiography (STE) is used to assess myocardial mechanics [12,13] and specifically LV strain. Left ventricular mechanics are complex and involve a combination of contraction/relaxation in different planes including longitudinal, circumferential, and radial orientations as well as twist and torsion [14,15,16]. Strain is usually derived from a 2D dataset [17,18], but advances in technology now allow the use of 3D echocardiography to determine real-time simultaneous strain from all planes of function [13]. There are currently no studies that have assessed LV structure and function using 3D echocardiography in physically active, healthy, and eumenorrheic females.

The resting 12-lead electrocardiogram (ECG) is used in clinical and research settings on new and established patients/participants to document heart rhythm abnormalities and to create a baseline assessment for future comparison. It is also used during pre-participation screening of young individuals for assessment and comparison across different time points. It is understood that compared to men, females typically have faster resting heart rates, longer QTc intervals, and shorter PR intervals and QRS duration, with sex hormones likely contributing, at least in part, to these differences [19]. However, the influence of the hormonal fluctuations across the menstrual cycle phases on the electrical activity is less clear. Previous research examining changes to the heart rate, interval durations, and wave amplitudes across the menstrual cycle phases has produced inconsistent results. The lack of consistency in the data could be attributed to heterogeneity in the study populations, lack of control of co-founding variables, or the lack of standardization of monitoring and reporting of the menstrual cycle phases. Conversely, arrhythmias such as supraventricular tachycardia have been shown to vary with the menstrual cycle and are more prevalent during the luteal phase when progesterone is at its highest and when higher levels of catecholamines are present [19,20,21]. Furthermore, regular endurance exercise training over the World Health Organisation-recommended 150 min exercise per week induces unique electrophysiological changes that must be considered when interpreting ECGs in this population [22]. There have not been any studies that have examined whether the menstrual cycle influences the resting ECG in healthy, physically active females. 

In view of the above considerations, the aim of this study was the following: (1) identify the impact of menstrual cycle phase (mid-luteal, ovulation, mid-follicular) on LV structure and function (including mechanics) using 3D echocardiography and (2) cardiac electrical activity (12-lead resting ECG) in healthy, physically active females. It was hypothesized that cardiac structure and function will be unchanged throughout the menstrual cycle phases and there would not be any significant changes in the ECG parameters. 

## 2. Methods

### 2.1. Participants 

Sixteen healthy female participants (20 y ± 2) volunteered to participate in the study. All participants engaged in a minimum of 2 h per week of physical activity and were instructed to maintain the same level of activity for the duration of the study. Participants were included if they had regular, pain-free menstrual cycles during the previous 12 months and had not taken oral contraceptives in the last 2 years. Participants were excluded if they were currently taking prescription drugs, were a smoker, or if they had a known cardiovascular disease, current symptoms suggestive of cardiovascular disease, family history of cardiovascular disease in a first-degree relative (<50 y), or a current illness. The study was approved by the Liverpool John Moores University Ethics Committee with all participants providing written informed consent.

### 2.2. Design

Participants attended the Cardiovascular Laboratory at Liverpool John Moore’s University for data collection at three time points during one menstrual cycle. Counting from the first day of menstruation, the measurements were assessed on day 7, day 14, and day 21 to capture the follicular phase, ovulation, and luteal phase, respectively. All visits occurred within ±1 day of these phases and occurred at the same time of day for each participant. All participants reported having a typical 28-day cycle. Participants were instructed to refrain from caffeinated beverages, alcohol consumption, and strenuous physical activity for at least 24 h before any testing.

### 2.3. Participant Evaluation

All participants completed a cardiac health questionnaire (personal symptoms, medical history, and family history of cardiovascular disease). Each session included anthropometrics (height (Seca Supra 719, Hannover, Germany); body mass (Seca 217, Hannover, Germany); calculated body surface area [23]; and body mass index (BMI) (kg/m^2^)), blood pressure, 12-lead resting ECG, and an echocardiogram. The participants’ brachial artery blood pressure was measured after five minutes of seated rest, a minimum of two times, using an automated sphygmomanometer (Dinamap 300, GE medical system, Orange City, FL, USA).

### 2.4. Echocardiography

The transthoracic echocardiographic examination was undertaken using a single experienced sonographer using a commercially available ultrasound system (Vivid E95, GE Healthcare, Horten, Norway). A real-time 3D echocardiographic image was acquired using the matrix-array transducer (4V-D) from the apical acoustic window. Image acquisition occurred during 4 cardiac cycles to ensure optimal spatial and temporal resolution at a frame rate of greater than 30 frames/second. A six-beat image acquisition was performed while the patient held their breath to eliminate breathing-related motion artifacts [13]. Analysis of the 3D echocardiographic images of LVEDV, LVESV, LV ejection fraction (LVEF), LVM, global longitudinal strain (GLS), global circumferential strain (GCS), global radial strain (GRS), and torsion was performed off-line using a software-specific semi-automated function image tool (EchoPac version 203, GE Medical, Horten, Norway). First, the alignment of the apical views was ensured to minimize foreshortening. Second, end-diastolic and end-systolic endocardial borders were defined by marking the LV apex and mitral annular plane. Automated definition of the LV endocardial border was performed with manual adjustment. After confirming the definition of the myocardial wall, strain analysis of all 17 segments was produced. This allowed peak GLS, GCS, GRS, and torsion to be measured. If more than three segments were missing from the analysis, then global strain values were unable to be calculated. Left ventricular concentricity was calculated as [LVM/LVEDV^2/3^]. Left ventricular volumes were indexed to BSA and LVM was indexed to height raised to the allometric power of 2.7 (LVMi) [24].

### 2.5. Electrocardiography 

All participants underwent a standard resting 12-lead ECG (CardioExpress SL6, Spacelab Health care, Snoqualmie, WA, USA) by a trained cardiac physiologist. The ECG was analyzed by a sports cardiologist. The ECG parameters included heart rate, P wave duration, PR interval, QRS duration, corrected (Bazett) QTc duration, and QRS axis. The values were evaluated according to established criteria [25]. 

## 3. Reproducibility Analysis

Three-dimensional echocardiographic images were analyzed three times by a blinded observer. Intra-observer reliability was assessed using an intraclass correlation coefficient (ICC). An ICC of 0.50 to 0.75 was considered moderate, an ICC of 0.75 to 0.90 was considered good, and >0.90 was considered excellent [26]. The intra-observer reliability for 3D parameters was rated as good to excellent, except for torsion, which was rated as moderate (Supplementary Material).

## 4. Statistical Analysis

All data are presented as mean ± SD. All data were tested for normality using the Shapiro–Wilk test. The data across all three visits were analyzed using a repeated-measures ANOVA and Bonferroni post-hoc test for multiple comparisons between the three menstrual phases using a commercially available software package, SPSS Version 29.0 for Windows (IBM Corporation, Somers, NY, USA). A probability value <0.05 was considered significant.

## 5. Results

Participants engaged in 5 ± 2 h per week of physical activity. Participant demographics are presented in Table 1. No significant differences were seen in weight, BMI, BSA, heart rate, or systolic blood pressure across the three phases of the menstrual cycle (*p* > 0.05). Diastolic blood pressure was lowest during the luteal phase (67 mmHg ± 8) compared to the follicular (73 mmHg ± 7) and ovulation (71 mmHg ± 8) phases (*p* = 0.015). 

**Figure 1:**
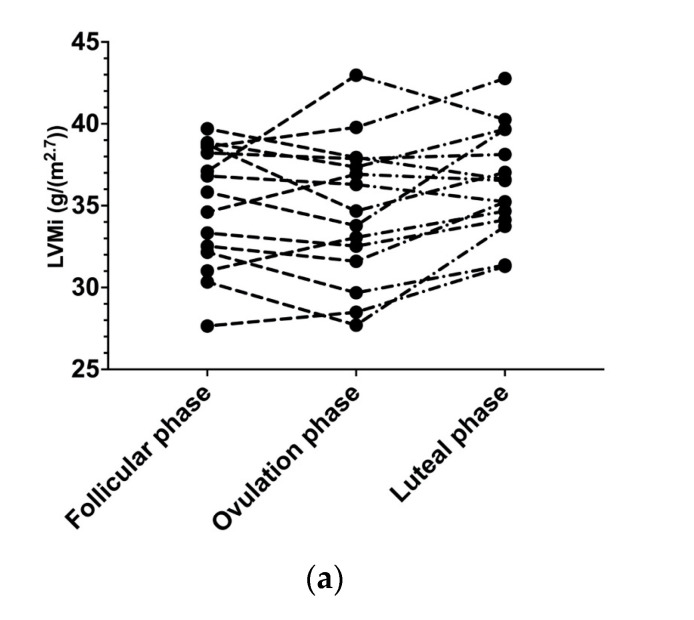

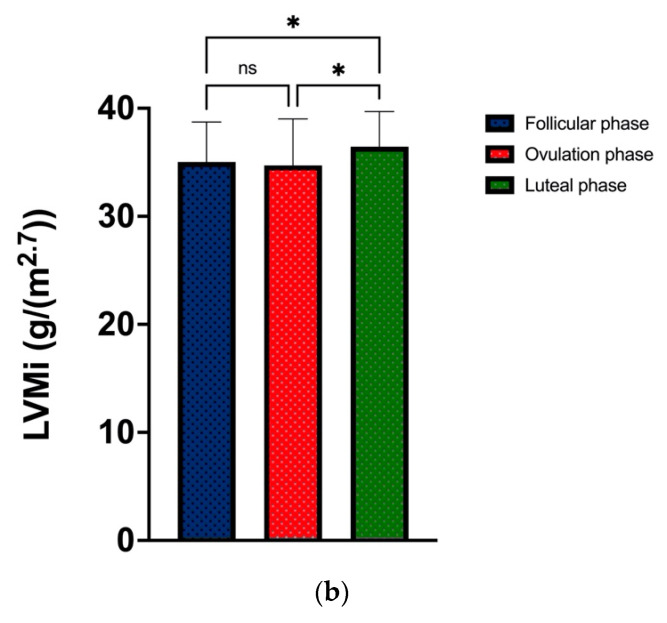
(**a**) Left ventricular mass indexed (LVMi) heterogeneity between subjects; (**b**) mean values of LVMi across the menstrual cycle phases. * *p* < 0.05; ns: non-significant.

**Figure 2:**
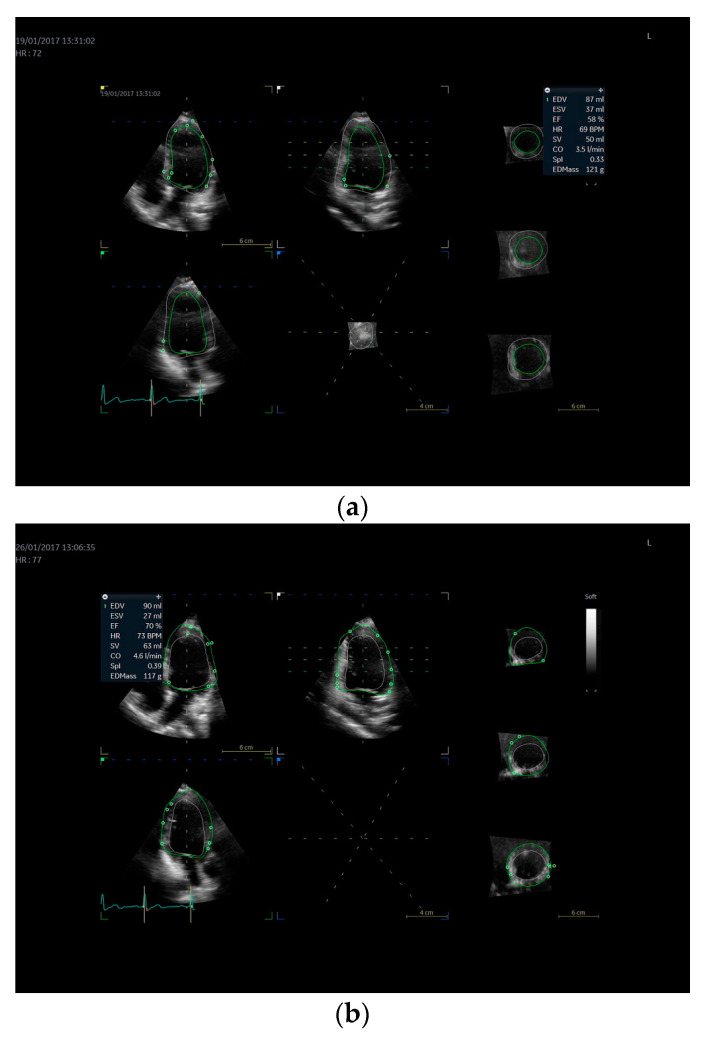

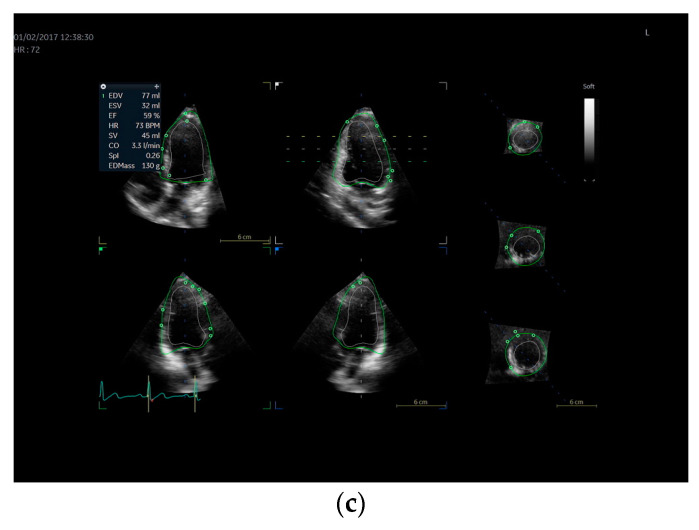
Exemplary 3D echocardiography images of a single participant with measurements made at (**a**) follicular, (**b**) ovulation, and (**c**) luteal phases of the menstrual cycle.

Detailed 3D echocardiographic characteristics of the participants are summarized in Table 2. One participant was excluded due to sub-optimal 3D images and insufficient image quality in any one of the data collection points. There was an increase in LVM during the luteal phase (147 g ± 22) compared to the follicular (142 g ± 25) and ovulation (140 g ± 23) phases (*p* = 0.032); however, pair-wise comparisons using the Bonferroni post-hoc test between the menstrual phases were not significant. When LVM was indexed allometrically for height, there was a significant increase during the luteal phase (36.4 g/m^2.7^ ± 3.3) compared to the follicular (35.0 g/m^2.7^ ± 3.7) and ovulation (34.7 g/m^2.7^ ± 4.3) phases (*p* = 0.026), albeit with some individual heterogeneity between subjects (Figure 1a,b). An exemplary case demonstrating LVM measurement using 3D echocardiography and the differences between the three menstrual phases is shown in Figure 2a–c. There were no significant differences across the phases for any other structural measures. All measures of LV mechanics and conventional functional indices were not different between phases of the menstrual cycle. 

The ECG parameters are presented in Table 3 with no significant differences in any of the indices across the menstrual cycle. One participant met the criteria for right axis deviation across all phases of the menstrual cycle. There were no arrhythmias, or atrial or ventricular ectopic beats observed. 

## 6. Discussion

The main findings from this study are as follows: (1) 3D-derived echocardiography LVMi is increased in the luteal phase compared to the other phases of the menstrual cycle and (2) no differences were observed across the menstrual cycle for other LV structural, functional, or electrophysiological indices in healthy, physically active females.

In previous studies that utilized M-mode and standard 2D echocardiography, there was a lack of any significant variation in LV structure (i.e., LVM) and function (i.e., LVEDV, LVESV, SV, LV ejection fraction) between the menstrual cycle phases [3,4,6]. Whilst the present study, using 3D echocardiography, similarly did not find a significant change in LV function, a change in LVMi was observed between the luteal phase and both the ovulation and follicular phases. Previous comparative studies have shown that 3D echocardiography-derived LVM values are smaller compared to 2D echocardiography-derived values and are similar to CMR values [27,28]. An important advantage of 3D echocardiography is the lack of reliance on geometric assumptions for determining LVM and LV volumes, and when combined with superior inter and intraobserver reliability, the modality may have greater sensitivity and ability to detect subtle changes, as demonstrated in the present study [29]. Indexed left ventricular mass was highest in the luteal phase and lowest in the ovulation phase when estrogen is at its lowest and highest, respectively. It is therefore possible that estrogen played a role in these differences; however, this cannot be confirmed with the present findings as the potential mechanisms for these differences were not examined. Despite the statistical significance across the menstrual cycle phases, LVMi elicited a heterogenous presentation among participants, which could be explained by the inter-individual fluctuations in hormone levels [30]. Previous research has demonstrated that in post-menopausal females—which is marked by a decrease in oestradiol (E2)—sex hormone binding globulin (SHBG) and testosterone demonstrate adverse LV remodeling [31]. These data highlight the association between LVM and hormones, and albeit much more acute and subtle in young eumenorrheic females, the decrease in estrogen during the luteal phase, which may in part contribute to differences observed in this study. It is important to note that these small, yet significant changes found using 3D echocardiography in the present young and healthy population are not likely of any clinical relevance as they are transient and not associated with deleterious changes in function. The present findings should be confirmed in future longitudinal studies with larger sample sizes.

A reduction in diastolic blood pressure was observed in the luteal phase compared to both the follicular and ovulation phases, and it would be conceivable to expect that the reduced afterload in this phase may result in concomitant changes in LV function. Since there was no adverse impact on LV function during the luteal phase, this drop in diastolic blood pressure can be regarded as a normal physiological phenomenon and likely attributed to the increase in progesterone and estrogen in this phase due to their vasodilatory effect [1]. Other studies have demonstrated small changes in diastolic function and systolic function derived from transmitral Doppler and tissue Doppler, respectively [4,9]. We did not directly assess diastolic function, but the comprehensive assessment of LV mechanics excluded any systolic changes and, due to the co-existing nature of GLS with diastolic dysfunction, it is likely that any differences would have been detected. Additionally, these studies did not detail specific loading conditions, which may have confounded their results knowing that tissue Doppler imaging and transmitral Doppler are more load dependent than absolute strain indices [4,9]. 

The present study did not observe any significant resting ECG changes or arrhythmias across the menstrual cycle. In previous research, the ECG has been used to analyze autonomic tone and the presence of arrhythmias to determine the effect of sex hormones during the menstrual cycle [4]. While a longer QT interval has been repeatedly observed in females compared to men, changes in the QT interval among the three phases of the menstrual cycle do not occur when assessed using a resting 12-lead ECG, which is confirmed by the present findings [32,33]. Additionally, long-term ECG monitoring to determine associations between hormone concentrations and the presence of supraventricular arrhythmias across the menstrual cycle phases has been used [34]. They observed a positive correlation between plasma concentrations of progesterone and the number and duration of supraventricular tachycardia [34]. In the present study, supraventricular arrhythmias were not observed, but it is important to note that the 12-lead ECG is a ten-second snapshot and does not provide long-term details as with long-term Holter monitoring. Additionally, we studied a young, healthy, and physically active population with no prior symptoms. The lack of systematic change in LV function using 3D echocardiography and a resting 12-lead ECG across the menstrual cycle provides greater confidence when interpreting differences or changes in observations following a research intervention or during a serial assessment. 

## 7. Limitations

Ovarian steroid levels were not directly measured and therefore biochemical confirmation (i.e., urinary luteinizing hormone) of the phase was not confirmed. Research has demonstrated that the assumption that each female has a repeated 28-day menstrual cycle with ovulation occurring on the same day is inaccurate [35]. The present study determined the menstrual cycle phase from the first day of the participants’ last menses and on the basis of a 28-day cycle, which all participants confirmed having. Since this study commenced, a call for more rigor and reproducibility in female cardiovascular research was published [36]. Future research should collect and report the details of the participants’ menstrual cycle [37], which will add further clarity to the present results. A further limitation of this study is that it did not examine the mechanisms behind what could cause the inter-individual variability in LVMi. There is also scope for 3D electrocardiographic mapping and machine learning within ECG analysis which may provide more sensitive data. We suggest that future studies consider this methodology. That aside, the 12-lead ECG was used in this study to highlight the clinical utility, and the interpretation of the findings has been made as such. This is the primary investigation in pre-participation screening, serial research assessments, and in patients with cardiac disease, and hence serves as the rationale for inclusion in this study. Finally, this study was conducted on healthy, physically active females, and therefore further research is required to establish the impact of the menstrual cycle on training status and in patients with disease. It is likely that this study is unpowered. However, the exploratory nature of this work still provides insight into the variation of these indices across the menstrual cycle. 

## 8. Conclusions

In physically active females, there is a small but significantly higher 3D echocardiographic-derived LVMi associated with the luteal phase of the menstrual cycle with no concomitant change in LV function or ECG parameters. These findings are important to consider when conducting clinical or research serial assessments, such that a small change in LVMi with no concomitant change in LV function or 12-lead ECG parameters likely has no clinical relevance.

## Figures and Tables

**Table 1:** Baseline demographics.

Parameter	Follicular Phase (Mean ± SD)	Ovulation Phase (Mean ± SD)	Luteal Phase (Mean ± SD)	*p*-Value
Age (y)	21 ± 2	-	-	-
Height (m)	1.67 ± 0.07	-	-	-
Weight (kg)	69 ± 11	69 ± 12	70 ± 12	0.160
BMI (kg/m^2^)	25 ± 4	25 ± 4	25 ± 4	0.450
BSA (m^2^)	1.8 ± 0.2	1.8 ± 0.2	1.8 ± 0.2	0.216
Systolic blood pressure (mmHg)	124 ± 11	119 ± 7	122 ± 11	0.401
Diastolic blood pressure (mmHg)	73 ± 7	71 ± 8	67 ± 8	0.015 *^

BMI: body mass index; BSA: body surface area. * significance between FP and LP. ^ significance between OP and LP.

**Table 2:** 3D echocardiographic data across menstrual phases.

Parameter	Follicular Phase (Mean ± SD)	Ovulation Phase (Mean ± SD)	Luteal Phase (Mean ± SD)	*p*-Value
LVEDV (mL)	105 ± 24	105 ± 26	108 ± 27	0.444
LVEDVi (mL/m^2^)	60 ± 14	58 ± 13	59 ± 13	0.681
LVESV (mL)	48 ± 10	49 ± 10	51 ± 11	0.194
LVEF (%)	54 ± 3	53 ± 3	52 ± 4	0.403
SV (mL)	57 ± 15	56 ± 16	57 ± 17	0.885
CO (mL)	3.7 ± 0.7	3.9 ± 0.9	3.9 ± 1.0	0.305
LVM (g)	142 ± 25	140 ± 23	147 ± 22	0.032 ^#^
LVMi (g/m^2.7^)	35.0 ± 3.7	34.7 ± 4.3	36.4 ± 3.3	0.026 *^
Concentricity (g/mL^2/3^)	6.4 ± 0.6	6.4 ± 0.6	6.6 ± 0.5	0.352
GLS (%)	−18.4 ± 2.1	−18.0 ± 2.6	−17.6 ± 4.8	0.796
GCS (%)	−15.8 ± 2.8	−16.8 ± 2.9	−17.2 ± 2.6	0.107
GRS (%)	47.7 ± 8.7	48.6 ± 7.8	52.0 ± 9.2	0.049 ^#^
Torsion (°)	9.5 ± 4.1	8.9 ± 3.9	7.9 ± 3.5	0.409

CO: cardiac output; GCS: global circumferential strain; GLS: global longitudinal strain; GRS: global radial strain; LVEDV: left ventricular end-diastolic volume; LVESV: left ventricular end-systolic volume; LVEF: left ventricular ejection fraction; SV: stroke volume; LVM: left ventricular mass; LVMi: left ventricular mass indexed. * significance between FP and LP. ^ significance between OP and LP. ^#^ Bonferroni post-hoc test was not significant.

**Table 3:** Electrocardiogram data across three menstrual cycle phases.

	FP Mean ± SD (Range)	OP Mean ± SD (Range)	LP Mean ± SD (Range)	*p*-Value
Heart rate (beats. min^−1^)	66 ± 16	73 ± 16	70 ± 15	0.062
P duration (ms)	100 ± 12	98 ± 9	98 ± 15	0.648
PR interval (ms)	152 ± 25	150 ± 22	149 ± 24	0.739
QRS duration (ms)	87 ± 9	88 ± 9	89 ± 10	0.236
QT corrected (Bazett) (ms)	415 ± 14	417 ± 15	411 ± 23	0.494
QRS axis (^o^)	63 ± 19	63 ± 25	66 ± 21	0.632

## Data Availability

The data underlying this article will be shared on reasonable request to the corresponding author.

## Supplementary Materials

The following supporting information can be downloaded at: https://www.mdpi.com/article/10.3390/jcdd10080331/s1, Table S1: 3D echocardiography reproducibility.
