# Supplementary material for: Three-Dimensional-Derived Echocardiographic Left Ventricular Structure and Function and Indices from the 12-Lead Electrocardiogram across the Menstrual Cycle in Healthy Physically Active Females: An Exploratory Study
Source: J Cardiovasc Dev Dis. 2023 Aug 3;10(8):331. doi: 10.3390/jcdd10080331 (PMC10455515; doi:10.3390/jcdd10080331)
Supplement: Supplementary file 1 [file jcdd-10-00331-s001.zip › jcdd-2509879-supplementary.pdf]

Table S1. 3D echocardiography reproducibility

Parameter	ICC (95% CI)	*p*-Value
LVEDV	0.992 (0.987 to 0.995)	<0.001
LVESV	0.942 (0.907 to 0.965)	<0.001
LVM	0.975 (0.960 to 0.985)	<0.001
GLS	0.847 (0.765 to 0.907)	<0.001
GCS	0.881 (0.815 to 0.928)	<0.001
GRS	0.895 (0.835 to 0.937)	<0.001
Torsion	0.637 (0.483 to 0.766)	<0.001
